# Four-dimensional *in vivo* X-ray microscopy with projection-guided gating

**DOI:** 10.1038/srep08727

**Published:** 2015-03-12

**Authors:** Rajmund Mokso, Daniel A. Schwyn, Simon M. Walker, Michael Doube, Martina Wicklein, Tonya Müller, Marco Stampanoni, Graham K. Taylor, Holger G. Krapp

**Affiliations:** 1 Department of Bioengineering, Imperial College London, UK; 2 Department of Zoology, University of Oxford, UK; 3 Swiss Light Source, Paul Scherrer Institut, Villigen, Switzerland; 4 Department of Comparative Biomedical Sciences, The Royal Veterinary College, London, UK; 5 Institute for Biomedical Engineering, ETH and University of Zurich, Switzerland

## Abstract

Visualizing fast micrometer scale internal movements of small animals is a key challenge for functional anatomy, physiology and biomechanics. We combine phase contrast tomographic microscopy (down to 3.3 μm voxel size) with retrospective, projection-based gating (in the order of hundreds of microseconds) to improve the spatiotemporal resolution by an order of magnitude over previous studies. We demonstrate our method by visualizing 20 three-dimensional snapshots through the 150 Hz oscillations of the blowfly flight motor.

Studies of animal movement are founded upon our ability to measure and model the underlying musculoskeletal mechanisms, and have long driven the advances of imaging techniques. In the late 19^th^ century, Muybridge's photographic time series of galloping horses provided the first visualizations of locomotor behaviors too fast to be resolved by the human eye^1^, presaging the age of cinematography. A century and a half on, modern motion-capture technology has made the imaging of fast external movements almost routine, and has also facilitated radiographic imaging of some internal movements^2^. X-rays penetrate tissues that are opaque to visible light^3^, and have been used extensively in visualizing vertebrate motion^4^. Radiographic imaging of live invertebrates has only become practical more recently, thanks to the very short exposure times and high spatial resolution permitted by the brilliance of X-rays of third-generation synchrotron light sources^5^,^7^. Radiographic time series of living insects have already led to deepened understanding of the mechanics of tracheal respiration^7^, and of pistoning motions in the gut of crawling caterpillars^8^. But, because they overlay all of the tissues in the X-ray's path, radiographic projections alone cannot describe complex three-dimensional movements. The four-dimensional (4D) reconstructions that this requires may instead be obtained through computed tomography (CT) using radiographic projections acquired from multiple viewing angles at multiple phases of the movement cycle^9^,^10^.

Non-repetitive motions can only be reconstructed in 3D if they occur on a timescale much longer than the time needed to obtain a complete set of angular projections^10^. However, for periodic or quasi-periodic movements, a three-dimensional image can be obtained by gating the projections at defined phases of multiple, consecutive movement cycles. The resulting tomograms do not represent a time-continuous description of a single period of movement, but instead represent a series of averages over multiple movement cycles. In cardiac clinical CT, for instance, radiographic acquisition is gated by the electrical activity of the heart as an external indicator of movement phase^11^. The same technique has been extended to image cardiac and respiratory movements in mice, achieving 12 ms temporal resolution and 150 μm voxel spacing in a clinical CT scanner^12^, and 17 ms temporal resolution and 20 μm voxel spacing at a synchrotron facility^13^. Impressive as these results are, each prospective or retrospective gating technique published so far relies on the acquisition of an external signal that is then used for gating. The movements of many animals, including those of most insects, fall beyond the reach of this approach.

To address this challenge, we developed a 4D synchrotron imaging technique combining single-exposure phase retrieval with retrospective gating. The resulting tomographic time series enable us to visualize the fast internal movements of the blowfly flight motor on sub-millisecond and micrometre scales. Said to be the most complex hinge in nature^14^,^15^, and containing more than 40 individual muscles oscillating at 150 Hz, the dipteran flight motor serves as a challenging target on which to demonstrate the potential applications of this technique to a range of biomechanical systems. In a first iteration of this technique^16^, we gated the projections retrospectively in a traditional way using an external indicator of the phase of the movement cycle (the fly's wingtip trajectory, filmed using a pair of high-speed cameras). Using an external gating signal is cumbersome, however, and may not always be possible. Hence, in order to generalize the method to other systems, we present here a refined technique that uses retrospective gating guided by the radiographic projections themselves, and provide a detailed validation of the performance of the method under various modes of data acquisition and analysis. Two sets of experiments were performed at the TOMCAT beamline^17^ of the Swiss Light Source. In the first set of experiments, we evaluated two different acquisition protocols using broadband radiation, imaging the anterior part of the flight motor, and the head-neck motor system. In the second set of experiments^16^, we imaged the complete flight motor, comparing monochromatic and broadband radiation. This allowed us to validate the robustness of retrospectively gated tomography to differences in experimental configuration. Synchrotron-based tomographic imaging requires the sample to be rotated quickly during radiographic acquisition, which could affect the behavior of a living sample in different ways depending upon rotation speed. To allow for an informed choice of this behaviorally significant parameter, we therefore explored the effects on image quality of using different rotation speeds.

## Results

**Figure 1:**
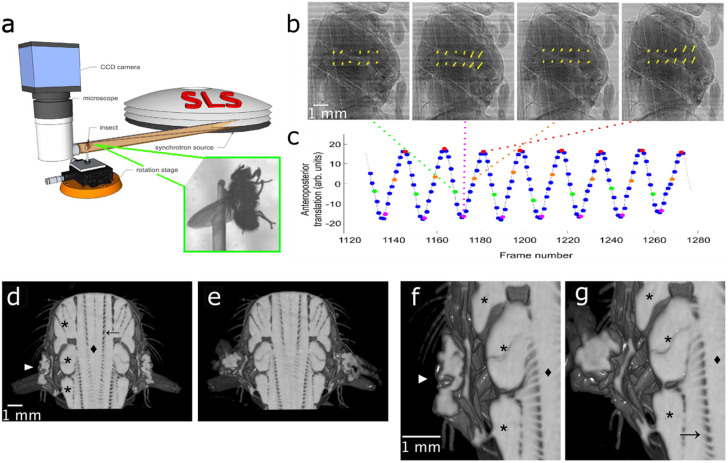
Experimental arrangement and retrospective gating. (a) The X-ray micro-tomography acquisition system (b) Normalized projections at four stages of one wingbeat cycle, with local image motion vectors (yellow arrows). The translation vectors were found by spatial cross-correlation of consecutive projections tracking the movements of twelve evenly spaced regions each containing 24 × 24 pixels. Orientation: head-neck joint at the top, posterior end of the thorax at the lower end of the images. Sequence of wingbeat phases from left to right: dorsal stroke reversal, downstroke, ventral stroke reversal, upstroke. (c) The gating signal (blue) derived from spatial cross-correlation of successive projections (after smoothing). The peaks of the signal (red points) identified the beginning of each new wing beat cycle, which was divided into uniformly spaced wingbeat phases. The green, pink, and orange points correspond to the phases of the wing beat shown in (b). (d–g) Rendering of the fly (d,e) thorax and (f,g) wing hinge (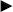) during (d,f) dorsal and (e,g) ventral stroke reversal (orientation: anterior towards the top, posterior towards the bottom). A frontal clipping plane has been introduced to reveal the large dorsal longitudinal and dorsoventral flight muscles, DLMs (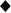) and DVMs (*), respectively. The ladder-like structure spanning the flight muscles is the tracheal network (→). At the level of the wing hinge, clear deformations of the exoskeleton are visible.

We began by acquiring radiographs of blowflies flying tethered to a rotating stage in single slow scan (SSS) mode (rotation speed: 54°s^−1^), single and multiple fast scan modes (SFS and MFS) (rotation speed: 332°s^−1^ or 347°s^−1^), and multiple medium scan mode (MMS) (rotation speed: 180°s^−1^), where a scan refers to a sample rotation of 180°. In the acquisition of multiple fast scans, we rotated the sample continuously. We compared the results obtained using monochromatic versus broadband (polychromatic) radiation. Single exposures of 0.2–0.4 ms and a total scanning time as short as 0.5 to 3.3 s allowed us to capture the fly spontaneously beating its wings with minimal motion artefacts. We retrieved the spatial distribution of the refractive indices of the tissues from the recorded phase deviation of the partially coherent synchrotron X-rays in the sample^18^. Compared to classical attenuation contrast, phase contrast enhances the visibility of tissue boundaries at a reduced radiation dose deposited in the specimen (Refs. 19,20, Methods). The projections from a single sequence were sorted according to the phase of the wingbeat cycle, using the spatial cross-correlation of each projection with the next to estimate the anteroposterior movement of the thorax between projections, which served as a retrospective gating signal (Fig.1b–c). Finally, for each of the 10 to 20 evenly spaced phase steps that we identified, we reconstructed tomographic sections through the blowfly thorax from approximately 500 projections. The wingbeat frequency of the blowflies was approximately 150 Hz, so the resulting tomographic time series have an effective temporal resolution of 1.5 to 3 kHz, depending upon scan mode.

We validated the performance of our method under each acquisition condition using standard image quality metrics (see Methods). The contrast between air and tissue was more than one order of magnitude higher in the reconstructed phase maps than it was in the attenuation-based reconstructions (Supplementary Fig. 1), although it remained difficult to distinguish between exoskeleton and soft tissue. Nevertheless, important functional elements of the fly's anatomy, such as the individual muscle units of the main flight muscles, were readily resolved at a spatial resolution of 9 to 31 μm, depending upon acquisition protocol (see Methods). The best overall scan quality was achieved using the ‘slow’ scan protocol with broadband radiation (spatial resolution: 10 μm; signal-to-noise ratio: 24 as detailed in the Methods). We validated our method by comparing its results to those of standard (i.e. non-gated) tomography of a fly at rest, acquired using monochromatic X-rays^20^. The spatial resolution of 15 μm and signal-to-noise ratio of 26 that we achieved using standard tomography were close to that of the retrospectively gated tomography (see Supplementary material). In conclusion, the two acquisition protocols, one involving radiographic acquisition during a single slow scan, and the other involving multiple ‘fast’ or ‘medium’ scans both have their own advantages and disadvantages. The lower sample rotation speed (54°s^−1^) that we used in the ‘slow’ scan diminished the perturbation experienced by the animal, but perhaps more importantly, it decreased the motion blur due to aperiodic movements and resulted in a more uniform sampling of the projection space. The fast rotation protocol, on the other hand, is likely to exhibit increased motion blur in this case, as well as strong streaking artefacts due to the uneven filling of the projection space^12^. Nevertheless, a pragmatic advantage of using multiple fast scans is that reconstructions will still be available, albeit of lower quality, if the animals stops moving before all scans are completed.

**Figure 2:**
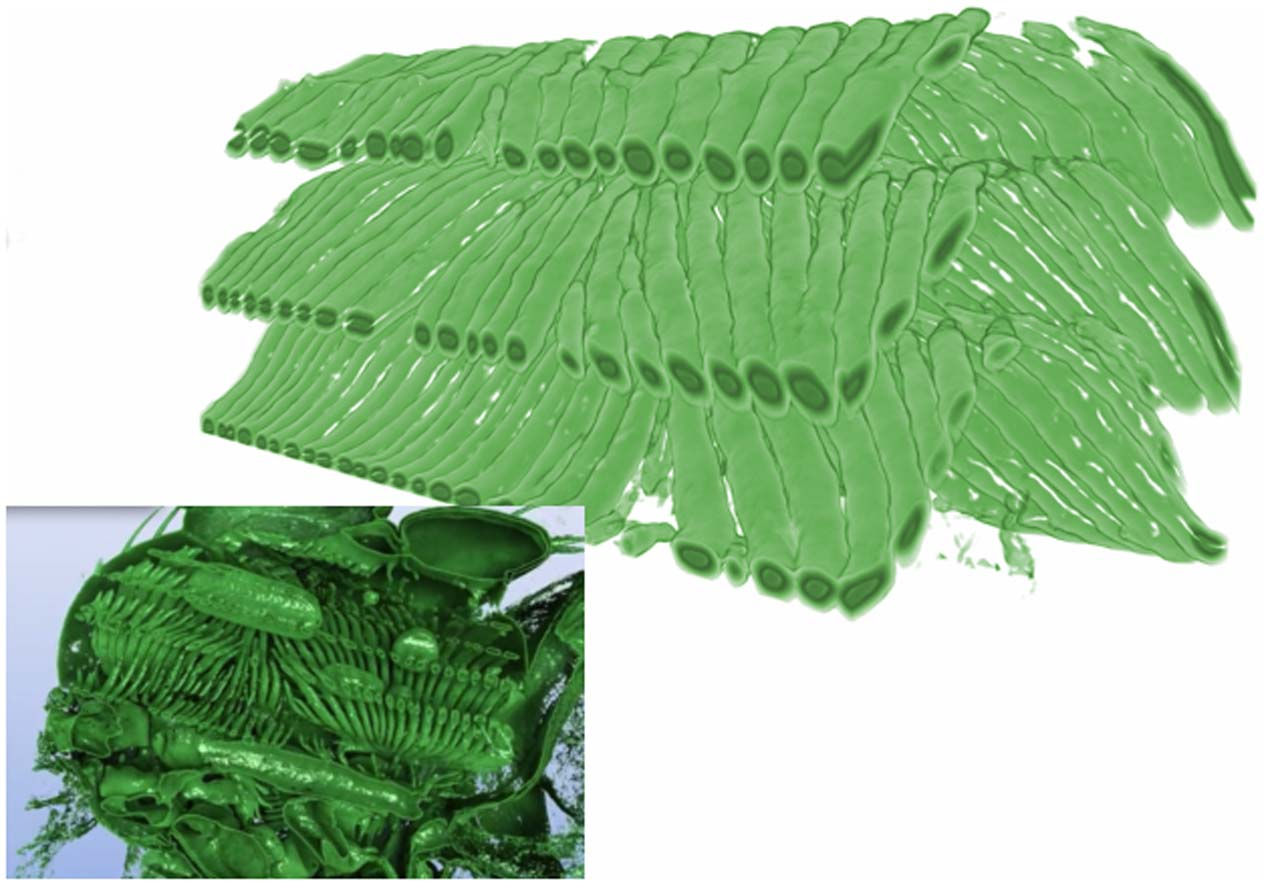
Visualization of the air-filled tracheal network spanning the dorsal longitudinal muscles (DLMs), and rendered in isolation from the rest of the thorax at the time of dorsal stroke reversal. The inset panel provides a similar rendering of the tracheal network within the rest of the thorax for context (see also Supplementary Movie 2). The large opaque green structures in the inset panel are air spaces inside the DLM.

As a visual demonstration of the quality of the data obtained, we used a reconstruction obtained using monochromatic X-rays at 18 and 15 keV in the fast and medium scan modes, respectively, to create volume renderings of the fly's dorsoventral and dorsal longitudinal flight muscles (DVM and DLM, respectively; Fig. 1 d–g). The six DVMs attach to the top and the bottom of the thorax, and contract alternately with the DLMs, which run along the anteroposterior axis of the thorax. Together the DVMs and DLMs power the wingbeat by causing the thorax to deform in a way that transmits force to the wing hinge. These muscles are prominent in the three-dimensional renderings of the thorax shown at ventral and dorsal stroke reversal (Fig. 1 d,e, Supplementary Video 2). Volume renderings of the wing hinge at the points of stroke reversal reveal the strong deformations of the cuticular exoskeleton that these muscles produce (Fig. 1 f,g). As a further demonstration, Fig. 2 shows the first ever 4D visualization of the tracheal network of a living insect. The different orders of tracheae in a blowfly cover diameters from hundreds of micrometers down to less than a micrometer; Fig. 2 shows branches with diameters between 20 and 50 μm. The major branches of the tracheal network separate the individual subunits of the DLMs, and can clearly be seen spanning the flight muscles. Animated renderings of the tracheal network (Supplementary Video 2,3) allow us to visualize the heaving motion of this complex structure at wingbeat frequency.

**Figure 3:**
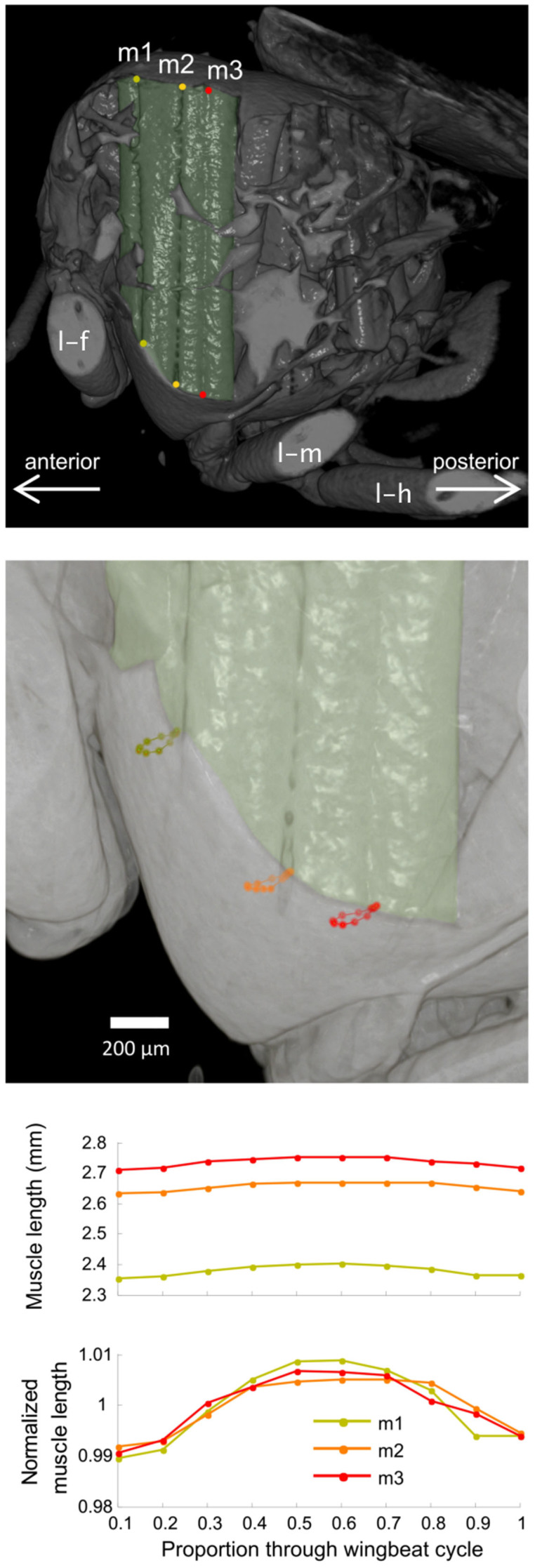
3D visualization of the flight muscles. (a) Rendering of dorsoventral muscle (DVM) with markers (wing beat stage: ventral stroke reversal). The dorsal longitudinal muscles (DLMs) are hidden behind other muscles in these visualizations. Three marker pairs were manually chosen in the region of the attachment site of the first unit of the DVM. The frontal (l-f), medial (l-m) and hind (l-h) legs are visible as is the attachment point of the fly holder (+). (b) Magnification of the ventral area of (a) with paths of the three markers at the ventral attachment site of DVM1. (c) Length oscillation and strain estimates of the first unit of the dorsoventral muscle (DVM1).

In Fig. 3(a,b) we show one frame from the volume renderings of the left, anterior DVM for ten equally-spaced phase steps through the wingbeat cycle. The section of cuticle covering the flank of the thorax is clipped away in the volume renderings to allow for an unobstructed view of the flight muscles. Thoracic deformations are clearly visible in the rendered time series, as are the contractions of the DVMs that drive these movements. The length of the left, anterior DVM was calculated at each time step for 3 pairs of virtual anatomical landmarks at the dorsal and ventral ends of the left DVM, corresponding to the intersections of the four subunits of the muscles (Fig. 3a,b). As a demonstration of the practicality of making automated measurements on these data, template matching was applied to measure the translational movement of each marker by maximizing the three-dimensional cross-correlation between a 25 × 25 × 25 voxel region around the marker between consecutive time steps (Fig. 3c). The length oscillations normalized by the average distance between a pair of markers were consistent between pairs, with the ventral markers moving up to 46.8 μm per time step. This movement mainly occurred in a plane almost normal to the muscle's dorsoventral axis. The maximum muscle strain was calculated by normalizing the peak-to-peak amplitude by the mean distance between the dorsal and ventral markers. The average muscle strain for the three marker pairs was 1.62% (standard deviation: 0.30%), which is consistent with videographic measurements of the displacements of external markers painted on the exoskeleton of fruit flies and observed during tethered flight^21^.

### Effects of radiation dose

The technique that we have developed here can be implemented using either broadband or monochromatic radiation. Flies exposed to either spectrum of X-rays (broadband or monochromatic) would typically stop flying within ten seconds of the start of the exposure, and later died, suggesting that lethal doses were incurred within seconds of the start of imaging. The estimated total dose^16^ was between 350 Gy and 1300 Gy: levels similar to those reported by Socha et al.^5^ to have no observable or permanent physiological effects, respectively, at lower incident beam fluxes. However, in our experiments, the dose was applied on a timescale of seconds rather than minutes, and the applied dose rates (between 90 and 325 Gy s^−1^) were one to two orders of magnitude higher than those applied in Ref. 5. Furthermore, whereas the insects in Ref. 5 were irradiated in a relatively inactive state, the insects in our experiments were performing the metabolically expensive action of flight. The flies' sensitivity to these high radiation rates is not unexpected^5^, but certainly merits further investigation.

## Discussion

In summary, using a projection-guided retrospective gating method, we were able to resolve and quantify the contractions of individual muscle sub-units powering the 150 Hz wing beat cycle of the blowfly, and were also able to visualize the volumetric changes of the tracheal network that ventilates and cools the flight motor. These results complement our earlier measurements of steering muscle strains, made using a similar technique where the wingtip kinematics were used to provide an external gating signal^16^. We achieved 4D reconstructions of the flight motor *in vivo* at 1.5–3 kHz effective temporal resolution with a voxel size of 3.3–5.0 μm, and corresponding true spatial resolution of 10–15 μm. This represents an order of magnitude improvement in spatiotemporal resolution as compared to previous studies. The technique that we have presented here is applicable in principle to any periodic biomechanical system that can be imaged successfully in a synchrotron. Considering its unmatched spatiotemporal resolution, the method that we have presented has the potential to revolutionize *in vivo* studies of functional anatomy, biomechanics, and physiology in small animals.

## Methods

### Preparation of specimens

Blowfly pupae (*Calliphora vicina*) were taken from the lab culture at the Department of Bioengineering, Imperial College London and kept on a 24 h (12:12) light-dark cycle. Specimens were used within two weeks of eclosion. They were briefly anesthetized for 8–10 minutes by cooling to 4°C, and fixed to a wooden tether at their scutum with a mixture of beeswax and collophonium. Care was taken to avoid the wax coming into contact with the scutellum. We fixed the wooden tether to a custom-made holder attached to the rotation stage of the beamline end station, and aligned the anteroposterior axis of the animal with the tomographic rotation axis. The results presented here were obtained from three specimens per acquisition mode (SFS reconstructions were based on a subset of projections from the MFS acquisition). Each specimen was scanned once.

### X-ray instrumentation and acquisition

The X-ray source was a superbending magnet located 25 m from the sample. For experiments with monochromatic light, a double crystal multilayer monochromator was used 7 m downstream of the source to extract monochromatic X-rays with a bandwidth of 2% at 18 and 15 keV photon energy (wavelength = 0.07 and 0.08 nm). The beam was 4.1 mm high and 10 mm wide at the sample position, and the flux at the sample was of order 10^12^ ph/s^1^/mm^2^ in this configuration. We selected a region of interest of 4.1 × 3.32 mm (1242 × 1008 pixels) to accommodate the whole thorax. The monochromator was omitted for experiments with broadband light, giving about 50 times greater total photon flux at the sample position and increasing the beam height to 5.7 mm. The broadband X-ray beam was filtered to 5% of its original power and accordingly the bandwidth was narrowed down around a peak value of about 35 keV (0.03 nm).

Radiographic projections were recorded at either 1840 Hz (0.4 ms exposure time) with broadband radiation, or at 2500 Hz (0.3 ms exposure time) or 4432 Hz (0.2 ms exposure time) with monochromatic radiation of 18 and 15 keV respectively, using a pco.Dimax 12 bit CMOS detector system. The detector was mounted on a custom made high numerical aperture microscope (Elya solutions, s.r.o.) featuring a continuous zoom-in option and a 100 μm thick LuAG:Ce scintillator crystal in its object plane for converting the X-ray photons to visible light photons that were collected by the CMOS sensor. The distance between sample and scintillator was 150 mm in broadband configuration, and 350 mm in monochromatic configuration. Isotropic voxel edge lengths were 3.3 μm (SSS, SFS, MFS) and 5.0 μm (MMS). These differences are the result of optimizing the sample to scintillator distance separately in each configuration: because we used a shorter single frame exposure time in the monochromatic configuration, a greater propagation distance provided better SNR as defined by Equation (1) after phase retrieval, with some accompanying loss in resolution.

### Phase retrieval

Previous studies^6^,^7^ conducted at partially coherent X-ray sources relied on the ‘edge-enhancement’ effect caused by interference of X-rays at the places where the refractive index has edges, and did not consider retrieving the actual phase shift of the probing X-rays in the sample. In phase retrieval experiments, ‘edge’-type contrast is transformed into ‘area’-type contrast, which provides segmentable images in 3D. We performed phase retrieval in a qualitative manner using our implementation of the single-image phase retrieval algorithm described previously^18^,^22^ under the assumption that the object consisted of a homogeneous soft tissue material. For the monochromatic beam (18 or 15 keV), we assumed that the refractive index decrements and absorption indices of the material were δ_18_ = 7 × 10^−7^ or δ_15_ = 9.9 × 10^−7^ and β_18_ = 5 × 10^−10^ or β_15_ = 9.8 × 10^−10^, respectively. For the broadband beam, we assumed that the mean x-ray energy was 35 keV and used values of δ_35_ = 2 × 10^−7^ and β_35_ = 10^−10^.

### Projection-guided retrospective gating and tomographic reconstruction

Each complete set of projections comprised 8350 radiographs taken for the angular range of 180° for the ‘slow’ rotation case (SSS), 4432 for the medium rotation speed (MMS) and 1000 or 1300 projections for the ‘fast’ rotation case (SFS, MFS). The first scan (i.e. 180° rotation) of an MFS acquisition was used for each SFS reconstruction. The fly beat its wings approximately 480 times during each 180° scan in the ‘slow’ rotation case, and approximately 80 times during each 180° scan in the ‘fast’ rotation case. Each 180° scan therefore sampled multiple wingbeats, and multiple stages of the wingbeat cycle. We developed projection-guided retrospective gating to group projections taken from different angles but at the same phase during the wing beat cycle. This is important in keeping experimental complexity to a minimum, by avoiding the need to identify and record a separate external gating signal.

The main movement of the animal's thorax during tethered flight was along the anteroposterior body axis. We therefore used the spatial cross-correlation of each projection with the next to estimate the vertical movement of the thorax between projections (Fig.1 b,c). The time course of these vertical translation estimates was smoothed by convolution with a discrete Gaussian filter (1.4 frames). We used the peaks in the resulting periodic signal to identify the beginning of each wingbeat cycle, and sorted projections taken at the same phases on this basis (Fig.1c). Visual inspection of the projection groups was used to verify the success of the algorithm in sorting the projections. Tomographic reconstruction was performed using a Fourier transform-based algorithm^23^.

### Estimation of scan quality using signal-to-noise ratio and resolution

The local spatial resolution was quantified using the edge response function at naturally occurring sharp edges in reconstructed slices of the object. Eight such edges from muscle tissue to air were chosen manually in the region of the dorsal longitudinal muscles. We fitted an error function to a short line profile of two pixels width, and identified the point spread function with a Gaussian function as described in Ref. 24. The full width at half maximum of the calculated point spread function served as a measure of the spatial resolution. This metric indicates the minimal distance between two point sources at which their images can still be distinguished.

A second criterion was employed to measure the global spatial resolution in twenty equally spaced transverse slices at the level of the anterior thorax. We applied the technique described by Modregger et al.^25^ to divide the power spectrum of the images into object image signal and process noise. The resolution was then defined as the critical wavelength at which the contribution of the noise-free image was equal to the contribution of the noise.

The signal-to-noise ratio (SNR) describes the distance of the object intensity distribution from the background intensity distribution and is closely related to the ability to automatically segment the image. SNR is defined as:
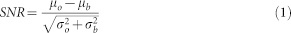
μ_o_ and μ_b_ are the mean intensity of patches of 12 × 12 object and background pixels, respectively, and where σ_o_ and σ_b_ are the standard deviation of pixel intensity in the same object and background patch. Twelve pairs of patches were chosen pseudo-randomly across the field of view in each of four slices, and for each of the data sets. We assumed that the true refractive index of the object represented by these patches was uniform.

Tomograms from 480 projections in the slow scan setup (Fig. 1) had a SNR of just 0.43 (±0.18 SD) if only attenuation and edge-enhancing phase contrast was reconstructed (Supplementary Fig. 1a). This was just sufficient to separate object and background voxels. Phasing fringes around the tissue-air-interfaces are visible as a halo-like edge enhancement effect in the attenuation-based reconstructions, indicating that there is additional information contained in the data that can be recovered through phase retrieval. Reconstructions obtained using phase retrieval (Supplementary Fig. 1b, Supplementary Video 1) had a much higher SNR, ranging from 16.2 (±5.7 SD) at worst (near the middle of the upstroke, when movement is fastest), to 17.8 (±4.4 SD) at best (at the end of the upstroke, when movement is slowest). The spatial resolution, defined as the full width at half maximum of the point spread function, was estimated at 16.2 (±2.4 SD) μm at worst (near the middle of the upstroke), and 14.3 (±2.0 SD) μm at best (at the end of the upstroke). The lowest spatial frequency that could still be resolved corresponded to 10.2 (±0.4 SD) μm at the end of the upstroke.

Tomographic sections reconstructed from 83 phase contrast projections acquired across a single fast scan with filtered broadband radiation (Supplementary Fig. 2a,b) exhibited a best-case SNR of 6.8 (±1.6). Combining the data acquired across six consecutive ‘fast’ scans of this fly gave a total of 507 projections (Supplementary Fig.2c,d) and improved the SNR to 15.7 (±2.9). The local spatial resolution in both cases (11.4 ± 1.3 μm and 15.4 ± 3.4 μm, respectively) was similar to that obtained with a single slow scan, as was the global spatial resolution (9.3 ± 0.2 μm and 10.3 ± 0.3 μm, respectively) (Supplementary Fig. 3). Visual inspection of the tomographic data revealed streaking artefacts in all of the data obtained from single and multiple fast scans (Supplementary Fig. 2a–d). These artefacts were not present in the data obtained from a single slow scan (Supplementary Fig. 1). Tomograms of the fly thorax acquired using monochromatic radiation at 18 keV (Supplementary Fig. 2e,f) had a higher SNR (23.5 ± 6.8) compared to the broadband case, but had somewhat decreased spatial resolution (31.4 ± 9.7 μm) as measured by the point spread function. These results are summarized in Supplementary Fig.3. The spatial resolution of gated tomographic imaging depends not only on the properties of the instrumentation used, but also on the consistency of the structure captured in different projections. This, in turn, is affected by the uniformity of the behavior or process under investigation, and by the movement velocity of parts of the specimen and the frame rate of the detector^26^. It is therefore not surprising that we obtained both good spatial resolution (13.5 ± 2) and SNR (16.3 ± 2.5) for the MMS (Supplementary Fig. 2g,h) with only 0.2 ms single exposure time and 15 keV monochromatic X-rays at a larger pixel size (5.0 μm instead of 3.3 μm).

## Author Contributions

D.A.S. and R.M. conceived this study. R.M. and M.S. designed the fast, synchrotron-based tomographic microscope. D.A.S., M.W., H.G.K., G.K.T., S.M.W., M.S. and R.M. designed the experiment. D.A.S., G.K.T., M.D., M.W., R.M., S.M.W. and T.M. performed research. D.A.S., M.D., R.M. and S.M.W. processed and analyzed data. D.A.S., R.M., H.G.K., M.S., S.M.W. and G.K.T. wrote the paper.

## Supplementary Material

Supplementary Information Tomographic slices through the flight muscle

Supplementary Information Tomographic reconstruction of a blowfly thorax

Supplementary Information Supplementary figures and captions

Supplementary Information The trachea network oscillation during one wingbeat cycle.

## References

[b1] Muybridge E. The science of the horse's motions. Sci Am 39, 241 (1878).

[b2] Owerkowicz T., Farmer C. G., Hicks J. W. & Brainerd E. L. Contribution of Gular Pumping to Lung Ventilation in Monitor Lizards. Science 284, 1661–1663 (1999). 10356394 10.1126/science.284.5420.1661

[b3] Socha J. J. & DeCarlo F. Use of synchrotron tomography to image naturalistic anatomy in insects. Proc SPIE 7078, 70780A (2008).

[b4] Jenkins F. A. & Dial K. P. A cineradiographic analysis of bird flight - the wishbone in starlings is a spring. Science 241, 1495–1498 (1988). 17790043 10.1126/science.241.4872.1495

[b5] Socha J. J., Westneat M. W., Harrison J. F., Waters J. S. & Lee W. K. Real-time phase-contrast x-ray imaging: a new technique for the study of animal form and function. BMC Biol 5, 6 (2007). 17331247 10.1186/1741-7007-5-6 PMC1831761

[b6] Betz O. *et al.* High-Speed X-ray Cineradiography for Analyzing Complex Kinematics in Living Insects. Synchrotron Radiation News 21, 37–41 (2008).

[b7] Westneat M. W. *et al.* Tracheal respiration in insects visualized with synchrotron x-ray imaging. Science 299, 52 (2003). 12543973 10.1126/science.1078008

[b8] Simon M. A. *et al.* Visceral-locomotory pistoning in crawling caterpillars. Curr Biol 20, 1458–63 (2010). 20655223 10.1016/j.cub.2010.06.059

[b9] Kalender W. A. X-ray computed tomography. Phys Med Biol 51, R29–43 (2006). 16790909 10.1088/0031-9155/51/13/R03

[b10] dos Santos Rolo T., Ershov A., van de Kamp T. & Baumbach T. In vivo X-ray cine-tomography for tracking morphological dynamics. PNAS 111, 3921–3926 (2014). 24594600 10.1073/pnas.1308650111 PMC3964127

[b11] Sagel S. *et al.* Gated computed tomography of the human heart. Invest Radiol 12, 563–566 (1977). 591261 10.1097/00004424-197711000-00019

[b12] Drangova M., Ford N. L., Detombe S. A., Wheatley A. R. & Holdsworth D. W. Fast retrospectively gated quantitative four-dimensional (4D) cardiac micro computed tomography imaging of free-breathing mice. Invest Radiol 42, 85–94 (2007). 17220726 10.1097/01.rli.0000251572.56139.a3

[b13] Dubsky S., Hooper S. B., Siu K. K. W. & Fouras A. Synchrotron-based dynamic computed tomography of tissue motion for regional lung function measurement. J R Soc Interface 9, 2213–2224 (2012). 22491972 10.1098/rsif.2012.0116 PMC3405755

[b14] Pringle J. W. S. Insect Flight. (Cambridge University Press, Cabridge, 1957).

[b15] Dickinson M. H. Insect Flight. Curr. Biol. 16 R310 (2006). 10.1016/j.cub.2006.03.087 16682333

[b16] Walker S. M. *et al.* In Vivo Time-Resolved Microtomography Reveals the Mechanics of the Blowfly Flight Motor. PLoS Biol. 12, e1001823 (2014) 24667677 10.1371/journal.pbio.1001823 PMC3965381

[b17] Mokso R., Marone F. & Stampanoni M. Real Time Tomography at the Swiss Light Source. AIP Conf Proc 1234, 87–90 (2010).

[b18] Paganin D., Mayo S. C., Gureyev T. E., Miller P. R. & Wilkins S. W. Simultaneous phase and amplitude extraction from a single defocused image of a homogeneous object. J Microsc 206, 33–40 (2002). 12000561 10.1046/j.1365-2818.2002.01010.x

[b19] Lovric G. *et al.* Dose Optimization Approach to Fast X-ray Microtomography of the Lung Alveoli. *J. Appl*. Cryst. 46, 856–860 (2013). 10.1107/S0021889813005591 PMC3769076 24046488

[b20] Mokso R. *et al.* Advantages of Phase Retrieval for Fast X-ray Tomographic Microscopy. J. Phys D 46, 494004 (2013).

[b21] Chan W. P. & Dickinson M. H. In vivo length oscillations of indirect flight muscles in the fruit fly Drosophila virilis. J Exp Biol 199, 2767–74 (1996). 9110958 10.1242/jeb.199.12.2767

[b22] Weitkamp T., Haas D., Wegrzynek D. & Rack A. ANKAphase: software for single-distance phase retrieval from inline X-ray phase-contrast radiographs. J Synch Rad 18, 617–29 (2011). 10.1107/S0909049511002895 21685680

[b23] Marone F., Münch B. & Stampanoni M. Fast reconstruction algorithm dealing with tomography artifacts. Proc SPIE 7804, 780410 (2010).

[b24] Bentzen S. M. Evaluation of the spatial resolution of a CT scanner by direct analysis of the edge response function. Med Phys 10, 579–81 (1983). 6646061 10.1118/1.595328

[b25] Modregger P., Lübbert D., Schäfer P. & Köhler R. Spatial resolution in Bragg-magnified X-ray images as determined by Fourier analysis. Phys Stat Sol (a) 204, 2746–2752 (2007).

[b26] Armitage S. E. J., Pollmann S. I., Detombe S. & Drangova M. Least-error projection sorting to optimize retrospectively gated cardiac micro-CT of free-breathing mice. Med Phys 39, 1452–61 (2012). 22380378 10.1118/1.3681949

