# Supplementary figures and images for: Four-dimensional in vivo X-ray microscopy with projection-guided gating
Source: Sci Rep. 2015 Mar 12;5:8727. doi: 10.1038/srep08727 (PMC4356984; doi:10.1038/srep08727)

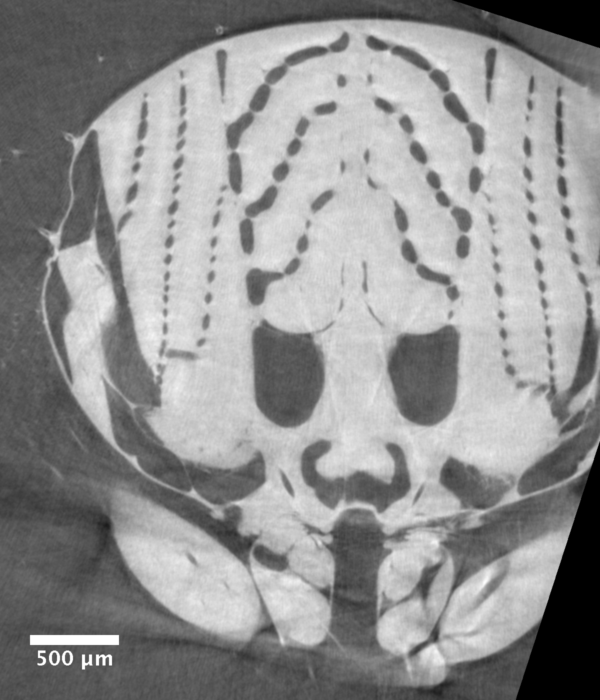

Supplement: Supplementary Information — Tomographic slices through the flight muscle [file srep08727-s1.gif]

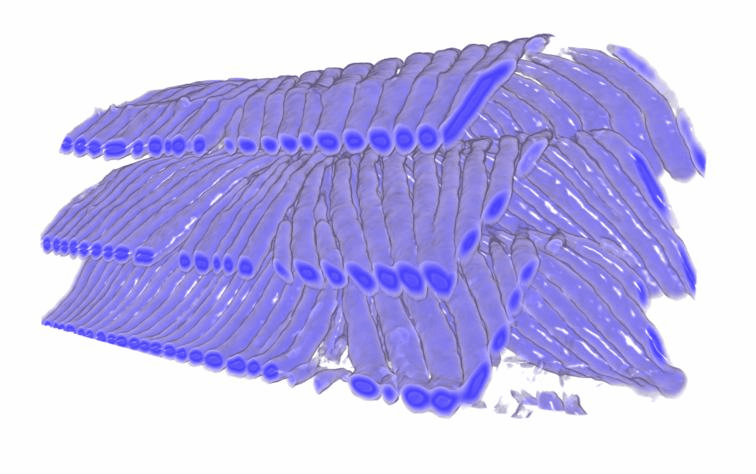

Supplement: Supplementary Information — The trachea network oscillation during one wingbeat cycle. [file srep08727-s4.gif]
