# Supplementary material for: Four-dimensional in vivo X-ray microscopy with projection-guided gating
Source: Sci Rep. 2015 Mar 12;5:8727. doi: 10.1038/srep08727 (PMC4356984; doi:10.1038/srep08727)
Supplement: Supplementary Information — Supplementary figures and captions [file srep08727-s3.pdf]

SUPPLEMENTARY INFORMATION:

Four-dimensional *in vivo* X-ray microscopy with projection-guided gating.

Rajmund Mokso[c,1,2], Daniel A. Schwyn[a,b,1], Simon M. Walker[b], Michael Doube[d], Martina Wicklein[a], Tonya Müller[b], Marco Stampanoni[c,e], Graham K. Taylor[b], Holger G. Krapp[a]

[a]Department of Bioengineering, Imperial College London, UK.
[b]Department of Zoology, University of Oxford, UK.
[c]Swiss Light Source, Paul Scherrer Institut, Villigen, Switzerland.
[d]Department of Comparative Biomedical Sciences, The Royal Veterinary College, London, UK.
[e]Institute for Biomedical Engineering, ETH and University of Zurich, Switzerland.
[1]These authors contributed equally to this study.
[2]To whom correspondence may be addressed. E-mail: rajmund.mokso@psi.ch

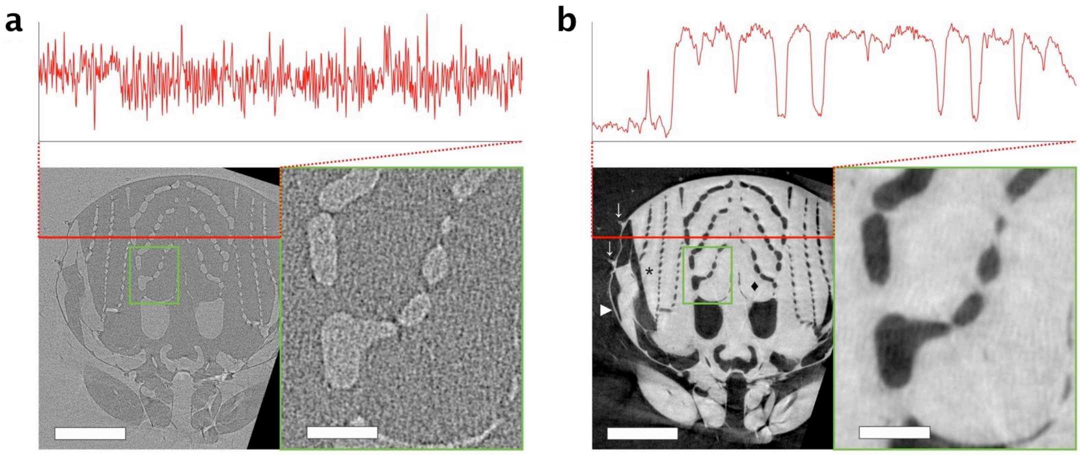

Supplementary Fig. 1
Quality of tomographic slices from a single slow scan. (a) Left: Reconstructed frontal section through the thorax at the level of the first pair of legs, obtained without using phase retrieval. The tomogram obtained from radiographs acquired in 3.3 s exhibits high spatial resolution and contrast, as well as minimal reconstruction and movement artefacts. Right: Five-fold magnification of the region of flight muscle outlined in green. Top: Intensity profile along the red line in the right image. (b) The same frontal section, reconstructed from phase-contrast radiographs. A cross section through the first dorsoventral muscle (*) and dorsal longitudinal muscle (♦), are distinctly visible, together with the cuticle of the exoskeleton (▶) and hairs (→). Right: Five-fold magnification of a region of flight muscle. Top: Intensity profile along the red line in the right image shows a higher ratio of signal to noise than the profile in A. Scale bars: Full slices (left): 1mm, magnified regions (right): 200 μm.

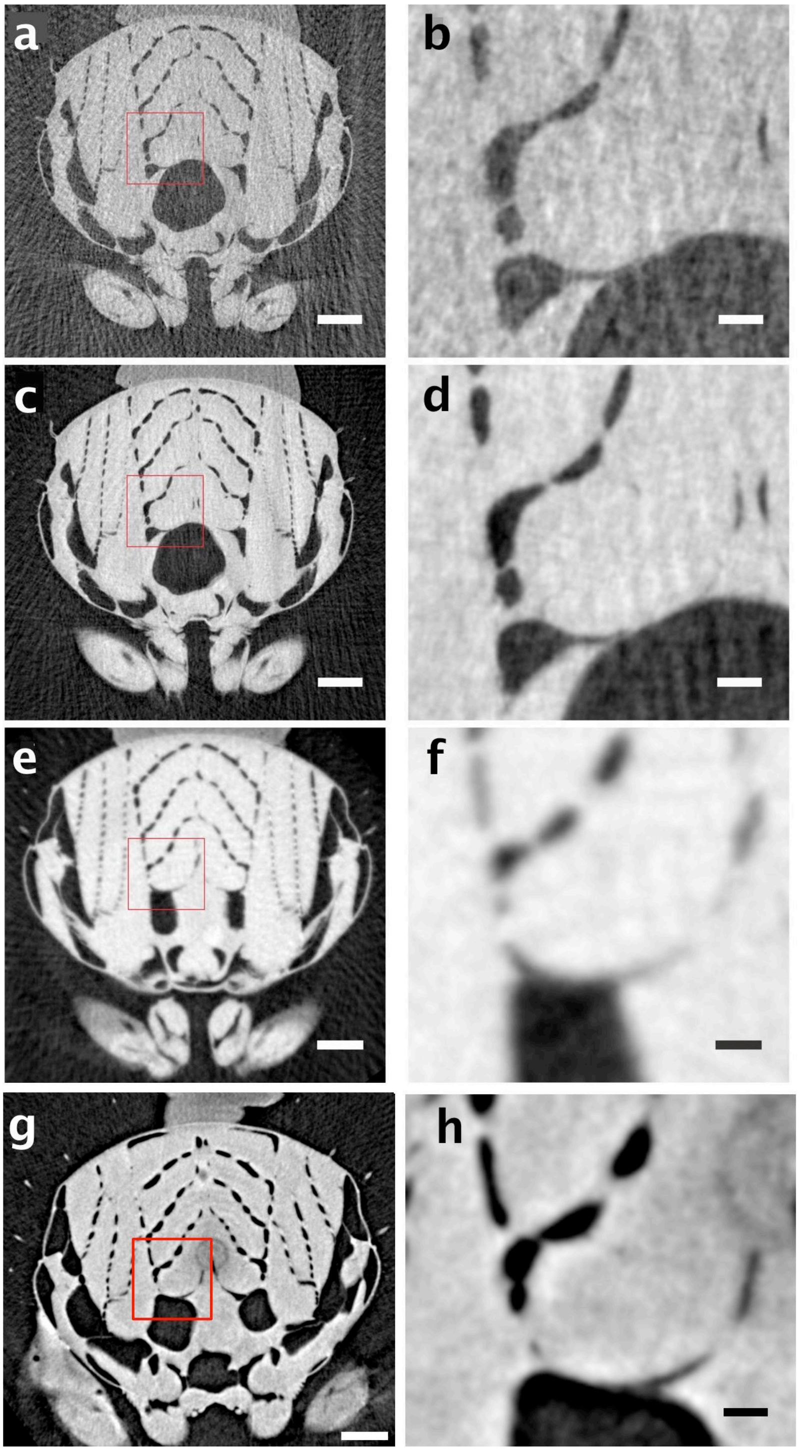

Supplementary Fig. 2

Dependence of image quality on acquisition protocol and radiation spectrum. Left column: Reconstructed cross sections through the thorax. Right column: Magnification of area indicated by red rectangle in left column. Tomographic slices in broadband X-ray configuration resulting from (a, b) a single fast scan (SFS) with total scan time of 0.5 s, exposure time of 0.4 ms and acquisition rate of 1840 Hz as compared to (c, d) six consecutive fast scans (MFS with total scan time of 3s). Tomographic slice in monochromatic (18 keV X-ray energy) configuration resulting from (e, f) multiple fast scans of a total scan time of 3.1 s. Tomographic slice in monochromatic 15 keV setup reconstructed from multiple medium scans (MMS) with total scan time of 4 s and an acquisition rate of 4432 Hz and exposure time of 0.2 ms. Scalebars: (a, c, e, g) 500 µm, (b, d, f, h) 200 µm.

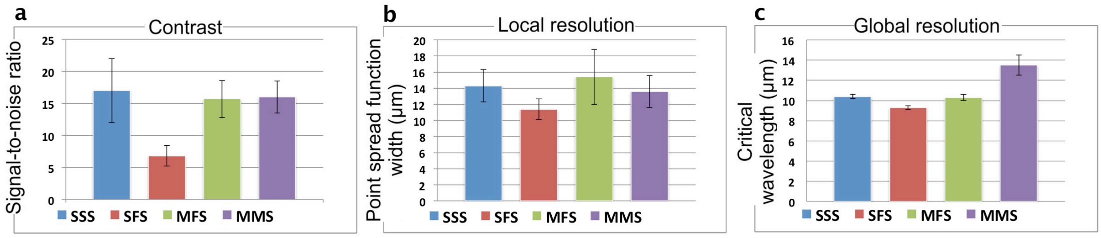

Supplementary Fig. 3
Image contrast and spatial resolution (mean ± SD) of tomographic reconstructions, evaluated for dorsal stroke reversal. (A) Tissue-air contrast was generally sufficient for automated segmentation, and higher for scans involving multiple rotations than for a single fast scan. (B) Local spatial resolution, as measured by the width (full width at half maximum) of the estimated point spread function, was comparable for all three protocols. Note that higher values indicate inferior resolution. (C) Global spatial resolution, as determined using a criterion based on the signal-to-noise ratio at different wavelengths, was on the order of 10 µm for all protocols. SSS: Single slow scan. SFS: Single fast scan. MFS: Multiple fast scans and MMS: Multiple medium scans.

Supplementary Video 1
Tomographic slices through the flight muscle for 10 phases of the wing beat. One wing beat cycle is visualized by selecting the same frontal section through the thorax at the level of the first pair of legs for each of the 10 time steps.

Supplementary Video 2
The tomographic reconstruction of a blowfly thorax created using the medium scan mode with twenty time steps, and highlights some of the features of the insect flight motor that are visible using this technique. At the start of the video the insect is facing to the left (the head is out of the field of view so appears clipped) and the attachment point of the mount on the scutum is visible at the top of the thorax.

As the view orbits the flying insect, the deformations of the thorax are clearly visible as the wing moves up and down, in particular the pitching and heaving motions of the scutellum (a bristle-covered structure posterior of the mount). Also visible are the beating halteres (dumbbell-shaped structures modified from the hindwings), which oscillate in a void covered by the calypteres (semi-transparent membranous structures, with an opaque border).

At 30s, a sagittal clipping plane is introduced. This first reveals the dorsoventral flight muscles (DVMs: coloured blue to purple) that power the wingbeat, along with the dorsolongitudinal flight muscles (DLMs: not coloured) that become visible as the clipping plane moves further into the thorax. An inverse threshold filter is then applied to reveal the tracheal network and other cavities within the flight muscle (shown in green).

The clipping plane then continues to move through the thorax until the steering muscles that provide fine control over the wingbeat are visible (coloured yellow to purple). The kinematics of the steering muscles and thoracic deformations are discussed in detail in (15), in which an external gating was used to gate the radiographs retrospectively and to correlate wing kinematics with muscle kinematics.

Supplementary Video 3
The trachea network oscillation during one wingbeat cycle. The threedimensional motion of the tracheal network in the dorsal longitudinal muscle is visualized for 10 time steps during one wing beat cycle. The air-filled spaces are rendered in semi-transparent blue; air spaces inside a region of the DLM in opaque blue.
